# Diagnostic imaging and cataloguing of female genital malformations

**DOI:** 10.1007/s13244-016-0515-4

**Published:** 2016-08-09

**Authors:** Pedro Acién, Maribel Acién

**Affiliations:** 1 Department of P.H., Sc.H. and Gynecology/Division of Gynecology, Miguel Hernández University, San Juan Campus, 03550 San Juan, Alicante Spain; 2 Obstetrics and Gynecology Service, San Juan University Hospital, San Juan, Spain; 3 Institute of Gynecology PAA, Alicante, Spain; 4 Departamento de Salud Pública, Historia de la Ciencia y Ginecología/Area de Ginecología, Facultad de Medicina de la Universidad “Miguel Hernández”, Campus de San Juan, 03550 Alicante, Spain

**Keywords:** Female genital malformations, Classification, Cataloguing, Diagnostic imaging, Complex malformations

## Abstract

**Abstract:**

To help physicians and radiologists in the diagnosis of female genito-urinary malformations, especially of complex cases, the embryology of the female genital tract, the basis for Müllerian development anomalies, the current classifications for such anomalies and the comparison for inclusion and cataloguing of female genital malformations are briefly reviewed. The use of the embryological system to catalogue female genito-urinary malformations may ultimately be more useful in correlations with clinical presentations and in helping with the appropriate diagnosis and treatment. Diagnostic imaging of the different genito-urinary anomalies are exposed, placing particular emphasis on the anomalies within group II of the embryological and clinical classification (distal mesonephric anomalies), all of them associated with unilateral renal agenesis or dysplasia. Similarly, emphasis is placed on cases of cervico-vaginal agenesis, cavitated noncommunicated uterine horns, and cloacal and urogenital sinus anomalies and malformative combinations, all of them complex malformations. Diagnostic imaging for all these anomalies is essential. The best imaging tools and when to evaluate for other anomalies are also analysed in this review.

***Teaching points*:**

*• The appropriate cataloguing of female genital malformations is controversial*.

*• An embryological classification system suggests the best diagnosis and appropriate management*.

*• The anomalies most frequently diagnosed incorrectly are the distal mesonephric anomalies (DMAs)*.

*• DMAs are associated with unilateral renal agenesis or renal dysplasia with ectopic ureter*.

*• We analyse other complex malformations. Diagnostic imaging for these anomalies is essential*.

## Introduction

It is important to identify abnormalities of the female reproductive tract as they are associated with a range of gynaecological and obstetric problems. Complex malformations, such as mesonephric and some Müllerian anomalies and also cloacal or urogenital sinus anomalies and malformative combinations, are especially important because in addition to creating fertility problems, they cause clinical symptoms and impact the quality of life, especially in young women. The overall prevalence of these disorders may be as high as 3 to 6 % and even higher in certain groups of women [1–3]. Today, there is increased detection caused by increased utility of imaging. The magnetic resonance image (MR) is the imaging standard of reference because it is non-invasive, does not involve ionising radiation, has multiplanar capability, allows excellent soft-tissue characterisation and permits a greater field of interrogation than ultrasound (US) (2D and 3D) [4–6]. However, other authors [7] believe that US (3D) could replace MR as the new gold imaging standard in diagnosing Müllerian anomalies.

Imaging and cataloguing of female genital malformations are important, but have the following prerequisites: (1) knowledge of the embryology of the female genito-urinary tract and interaction between the Wolffian/Müllerian ductal systems; (2) knowledge of anomalies involved in the classical Müllerian development as well as the septum resorption processes. Thus, to alert and help the physicians, especially radiologists, in diagnosing female genito-urinary malformations, these mentioned aspects will be reviewed briefly as well as the clinical presentation, catalogation and inclusion of female genital malformations in the embryological and clinical classification [8] and in other current classification systems. Finally, diagnostic imaging for all female genito-urinary malformations is presented with emphasis on the more complex anomalies, which are better understood on this embryologic basis, in other words, according to the updated embryological and clinical classification of female genito-urinary malformations [8].

### Embryology

**Fig. 1:**
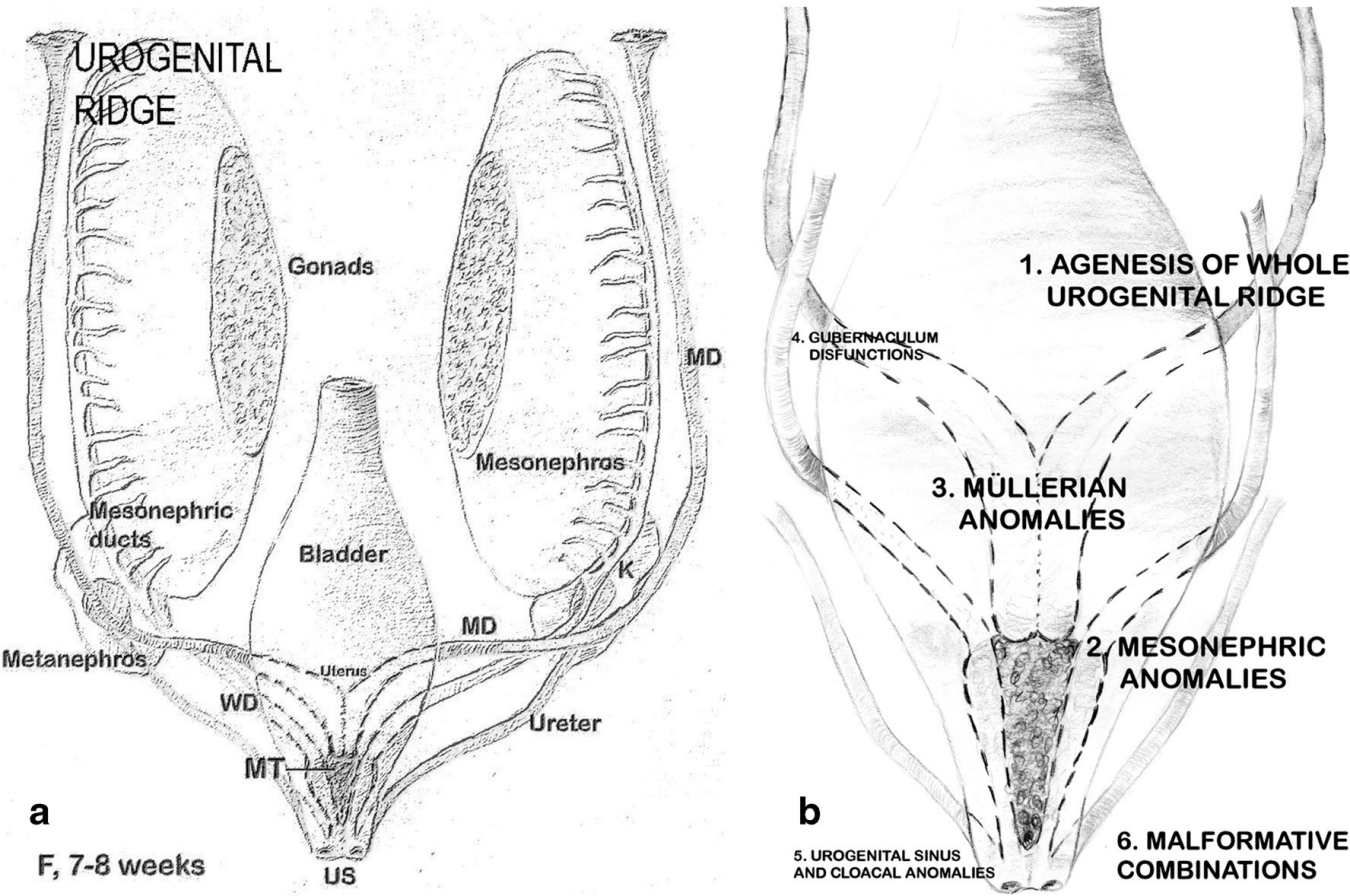
Embryology of the female genito-urinary tract. **a** Development of the genital ducts in the female (frontal view, 7–8 weeks). The formation of the uterine primordium and the opening of the mesonephric ducts into the urogenital sinus are shown. The Müllerian tubercle can be seen between both Wolffian ducts and the ureteral buds sprouting from the opening of the Wolffian duct into the urogenital sinus. MD, Müllerian ducts; WD, Wolffian ducts; K, kidney; MT, Müllerian tubercle; US, urogenital sinus. **b** On a diagram of the embryology of the female genital tract, the places and suggested pathogenesis for the origin of the different groups of malformations included in the embryological and clinical classification [8, 10] are shown

Figure 1 shows schemes of female genito-urinary embryology [8–11]. Briefly, the uterus is formed from the fusion of the distal segments of Müller’s ducts and the later reabsorption of the intermediate wall, whereas the vagina proceeds from the Wolffian ducts and Müllerian tubercle [9, 11]. The appropriate development, fusion and resorption of the wall that separates both Müller ducts are induced by the Wolffian ducts located at both sides, which act as guide elements. Moreover, since the ureteral bud sprouts from the opening of the Wolffian duct into the urogenital sinus, the absence or distal injury of one of these ducts will give rise to renal agenesis, ipsilateral blind or atretic hemivagina and a uterine anomaly (fusion or resorption defect). Other embryological considerations can be seen in different articles [8–15]. *Müllerian development anomalies*

In terms of the classical Müllerian development processes, it is important to distinguish the following:

1. Anomalies caused by total or partial agenesis of one (unicornuate uterus) or both Müllerian ducts [Mayer-Rokitansky-Kuster-Hauser (MRKH or Rokitansky syndrome].
2. Anomalies caused by total or partial absence of fusion (didelphys uterus and bicornuate-bicollis and unicollis-uterus).
3. Anomalies caused by total or partial absence of reabsorption of the septum between the Müllerian ducts (septate and subseptate uterus).
4. Anomalies caused by a lack of later development [hypoplastic uterus, T-shaped and diethylstilbestrol exposure (DES) syndrome] [15].

This classification system for uterine malformations is followed by the traditional classifications [5, 16–20] and the most recent cataloguing systems [21, 22]. However, several published cases showing a septate uterus with double cervix and vagina and normal uterus with septate cervix and vagina [23–25] questioned the classic hypothesis of unidirectional Müllerian development and supported the alternative embryologic hypothesis of Müller et al. [26], which states that fusion and resorption begin at the isthmus and proceed simultaneously in both the cranial and caudal directions.

These reported cases [23–25] and others [14] appear to prove the existence of a possible discrepancy in the processes of fusion and resorption between the superior-convergent and the inferior-divergent portions of the Müllerian ducts. Therefore, malformations can range from the didelphys-unicollis uterus to the bicervical normal uterus or normal uterus with a septate cervix and/or vagina [8, 14, 27]. The latest ESHRE/ESGE classification system “UCV” [21, 22] is also based on these Müllerian development processes, but considers uterine, cervical and vaginal anomalies, with anatomy being the basis for the systematic categorisation of female genital malformations.

## Comparison for inclusion and cataloguing of female genital malformations

The main classification systems for genital malformations refer to only Müllerian anomalies or the anatomic visual appearance and do not explain or suggest the actual origin of female genito-urinary tract malformations or their appropriate therapeutic correction. However, the embryological and clinical classification [8–10] correlates better among vaginal anomaly, uterine anomaly, and ipsilateral renal agenesis or renal dysplasia with or without ectopic ureter, suggesting the origin and possible clinical presentation and thus leading the diagnostic imaging.

Table 1 shows the congenital malformations of the female genito-urinary tract, their clinical presentation and cataloguing with the embryological and clinical classification [8] and also with the current classification systems [19, 22].

**Table 1:** Congenital malformations of the female genito-urinary tract, their inclusion in the embryological and clinical classification (Acién and Acién, 2011) and in other classification systems of female genital malformations (AFS/ASRM, 1988; ESHRE/ESGE, 2013) and clinical presentation

Congenital malformations of the female genito-urinary tract	As included in the embryological and clinical classification (Hum reprod update 2011;17/5:693–705)	As included in the AFS/ASRM classification of Müllerian anomalies (Fertil Steril 1988;49/6:944–55)	As included in the new ESHRE/ESGE classification system of female genital anomalies (Hum Reprod 2013;28/8:2032–44)	Clinical presentation
*1. Agenesis or hypoplasia of one urogenital ridge including unicornuate uterus with contralateral RA and the atypical Rokitansky syndrome*.	Group I: I.1. Rokitansky syndrome with URA (if contralateral Müllerian agenesis) I.2. Unicornuate uterus with contralateral RA	Class Ie (utero-vaginal agenesis). Additional findings: URA. Class II (unicornuate uterus). Additional findings: URA	U5 (aplastic)/C4 (cervical aplasia)/V4 (vaginal aplasia). Associated non-Müllerian anomalies: URA. U4 (hemiuterus)/C0/V0. Associated anomalies: URA	Primary amenorrhoea No symptoms. Reproductive. Breech
*2. Distal mesonephric anomalies, including URA and ipsilateral blind or atretic hemivagina syndrome*, showing:	Group II: All distal mesonephric anomalies: Uterine duplicity with blind hemivagina (or atresia) and URA (sometimes ectopic ureter and renal dysplasia or other ipsilateral renal anomalies)	Class III, IV or V (didelphus, bicornuate or septate uterus). Additional findings: vagina, cervix, kidneys	U3 or U2 (bicorporeal or septate uterus)/C1, C2 or C3 (septate, double or unilateral cervical aplasia)/V2, V1 or V0 (obstructing, non-obstructing vaginal septum or normal vagina). Associated non-Müllerian anomalies: URA, ectopic ureter	Girl, adolescent or young women presenting:
*2A. Obstructed or blind hemivagina with large haematocolpos (Wunderlich syndrome)*.	II.1 Didelphys or bicornuate (rarely septate) uterus with blind hemivagina and ipsilateral RA (sometimes ectopic ureter and renal dysplasia or other ipsilateral renal anomalies)	Class III, IV or V (didelphus, bicornuate or septate uterus). Additional findings: vagina, cervix, kidneys	U3 or U2 (bicorporeal or septate uterus)/C2, C1 (double, or septate cervix)/V2 (longitudinal obstructing vaginal septum). Associated non-Müllerian anomalies: URA, ectopic ureter	Pelvic pain. Acute urinary retention. Intra- and postmestrual dysmenorrhoea. Pelvic cystic mass. Postmenstrual spotting
*2B. A Gartner duct pseudocyst in the upper anterolateral wall of the vagina (Herlyn-Werner syndrome)*.	II.2 Bicornuate communicating uterus with athretic blind hemivagina and ipsilateral RA (sometimes ectopic ureter or mesonephric remnants)	Class IVb (partial bicornuate uterus). Additional findings: vagina, cervix, kidneys	U3a (partial bicorporeal uterus)/C3 (unilateral cervical aplasia)/V2 (longitudinal obstructing vaginal septum)^a^. Associated non-Müllerian anomalies: URA, ectopic ureter	Pain? Cysttic mass in anterolateral wall of vagina. Postmenstrual spotting or coital-related vaginal discharge
*2C. A short vaginal septum or a communicating buttonhole*	II.3 Didelphys or bicornis-bicollis uterus with a short vaginal septum or buttonhole due to partial reabsorption of the intervaginal septum and URA	Class III or IVa (didelphus or bicornuate uterus). Additional findings: vagina, cervix, kidneys	U3b, U3c (bicorporeal uterus)/C2 (double ‘normal’cervix)/V1 (longitudinal non-obstructing vaginal septum. Associated non-Müllerian anomalies: URA, ectopic ureter	No symptoms. Dyspareunia. Reproductive. Breech presentations. Obstetrical complications
2D. *Bicornuate-unicollis communicating uterus with with an anomalous horn and ipsilateral URA*	II.4 Bicornis-unicollis communicating uterus with unilateral cervicovaginal atresia and ipsilateral RA	Class IVb (partial bicornuate uterus). Additional findings: URA	U3a (partial bicorporeal uterus)/C3 (unilateral cervical aplasia)/V0 (normal vagina)^b^. Associated non-Müllerian anomalies: URA	No symptoms. Reproductive. breech presentation. Obstetrical compluications
2E. *Didelphys or unicornuate uterus with unattached and cavitated rudimentary horn, unilateral cervicovaginal atresia and ipsilateral URA*	II.5 Didelphys (ultrasound, MR) or unicornuate uterus with contralateral unattached and cavitated rudimentary horn, unilateral cervicovaginal atresia and ipsilateral URA	Class III (didelphus) or IIb (unicornuate uterus, non-communicating). Additional findings: URA	U3b or U4a (complete bicorporeal uterus)/C3 (unilateral cervical aplasia)/V0 (normal vagina)^c^. Associated non-Müllerian anomalies: URA	Pain. Symptoms as endometriosis. Endometriomas. Increasing dysmenorrhoea after surgery, adnexectomy
3. *Isolated Müllerian anomalies (without urinary tract anomalies)*	Group III. Isolated Müllerian anomalies affecting the ducts, tubercle or both elements	Class I to class VII	Class U1 to Class U5/C0, C1, C2, C4/V0, V1, V3, V4	Common uterine or uterovaginal anomalies.
3A. *Müllerian agenesis*, including typical *Rokitansky syndrome (sometimes with a cavitated rudimentary horn)*	III.A1,C. Müllerian agenesis and complete uterovaginal agenesis, Rokitansky or MRKH syndrome. Sometimes with a cavitated rudimentary horn	Class I. Hypoplasias/agenesis: vagina, cervical, fundal, tubal and combined	U5 [Aplastic uterus (a) with a rudimentary cavity or (b) without a rudimentary cavity]/C4 (cervical aplasia)/V4 (vaginal aplasia)	Primary amenorrhoea. Difficulty with sexual intercourse or infertility. Eventual endometriosis and cryptomenorrhoea
3B. *Unicornuate uterus (sometimes with cavitated non-communicating uterine horn; then externally bicornuated and sometimes septated)*	III.A2. Unicornuate uterus (or externally bicornuated) with atretic cavitated or non-cavitated rudimentary horn, or segmentary atresia or ‘unilateral Rokitansky’	Class II. Unicornuate. (a) communicating, (b) non-communicating, (c) no cavity, (d) no horn	U4 [hemiuterus (a) with a rudimentary cavity, communicating or not, or (b) without a rudimentary cavity or no horn]/C0/V0	Reproductive. Breech presentation. Intra- or postmenstrual dysmenorrhoea. Pelvic pain. Endometriosis?
3C. *Didelphys uterus (generally with double cervix and vagina)*	III.A3. Didelphys uterus	Class III. Didelphus	U3 (complete bicorporeal uterus)/C2 (double ‘normal’ cervix)/V1 (longitudinal non-obstructing vaginal septum)	Dyspareunia? Reproductive. Breech presentation
3D. *Bicornuate uterus (eventually with a non-communicating cavitated uterine horn)*	III.A4. Bicornuate uterus: bicornis-bicollis uterus and bicornis-unicollis uterus	Class IV. Bicornuate: (a) complete; (b) partial	U3 [bicorporeal uterus: (a) partial, (b) complete, (c) bicorporeal septate]/C0, C1, C2/V0, V1	Reproductive losses. Breech presentation Retrograde menstruation?
*3E. Septate uterus (eventually with a non-communicating cavitated uterine horn, Robert’s uterus)*	III.A5. Septate and subseptate uterus	Class V. Septate: (a) complete, (b) partial	U2 [septate uterus: (a) partial, (b) complete]/C0, C1, C2/V0, V1	Reproductive losses. Breech Eventually hemihaematometra?
3 F. *Arcuate* and hypoplastic uterus *(including DES syndrome and tricavitated uterus)*	III.A6. Arcuate uterus III.A7. Anomalies related to DES syndrome. Hypoplastic, T-shaped and tricavitated uterus	Class VI. Arcuate VII. DES drug related	U1 [dysmorphic uterus: (a) T-shaped, (b) infantilis, (c) others]/C0/V0	Reproductive losses? Infertility
3G. *Complete vaginal or cervico-vaginal atresia with normal uterus*	III.B1. Anomalies affecting Müllerian tubercle: Complete vaginal or cervico-vaginal agenesis or atresia	Class I. Hypoplasis/agenesis: (a) vaginal, (b) cervical	U0 (normal uterus)/C4 (cervical aplasia)/V4 (vaginal aplasia)	Primary amenorrea. Pelvic pain. Cryptomenorrhoea. Endometriosis
3H. *Transverse vaginal septum*	III.B2. Segmentary atresias. Complete or incomplete transverse vaginal septum	Not included Additional findings: vagina	U0/C0/V3 (transverse vaginal septum and/or imperforate hymen)	Primary amenorrhoea and cryptomenorrhoea. Pelvic pain, haematocolpos. Dyspareunia?, Obstetrical problems?
*4. Accesory and cavitated uterine masses with normal uterus (ACUMs)*	IV. Accesory and cavitated uterine masses and other gubernaculum dysfunctions	Not included	Not included	Pelvic pain. Severe dysmenorrhoea from menarche. Tumour?
5. *Anomalies of the urogenital sinus*	Group V. Anomalies of the cloaca and urogenital sinus Congenital vesico-vaginal fistula. Cloacal exstrophy.	Not included	Not included	
5A. *Imperforate hymen*	V.1. Imperforated hymen	Not included	U0/C0/V3 (transverse vaginal septum and/or imperforate hymen)	Primary amenorrhoea. Cryptomenorrea. Pelvic pain. Haematocolpos
5B. *Congenital vesico-vaginal or vagino-vesical fistula* (pseudo-lower vagina atresia)	V.2. Congenital vesico-vaginal fistula	Not included	Not included or U0/C0/V4 (vaginal aplasia)	Cyclical menuria Urinary incontinence? Pain. Dyspareunia. Hypospadias. Vaginal atresia
5C. *Cloacal exstrophy*	V.3. Cloacal anomalies. Persistent urogenital sinus	Not included	Not included	Generally paediatric patients. Urinary symptoms and incontinence. Extragenital associated anomalies
6. *Malformative combinations*	Group VI. Malformative combinations	Not included	U6 (unclassified anomalies). Associated non-Müllerian anomalies	Variable

AFS/ASRM, American Fertility Society/American Society for Reproductive Medicine; ESHRE/ESGE, European Society for Human Reproduction and Embryology/European Society for Gynaecological Endoscopy; MRKH, Mayer-Rokitansky-Kuster Hauser; MR, magnetic resonance; URA, unilateral renal agenesis. RA, renal agenesis. U, uterus; C, cervix; V, vagina. ^a^ It could initially be catalogued as U3a/C0/V0. ^b^ It could initially be catalogued as U3a/C0/V0 except for the suggestion from intravenous pyelography and performance of a hysterosalpingography and/or magnetic resonance. ^c^. It could initially be catalogued as U3b/C0/V0 or U4a/C0/V0

## Diagnostic imaging

Based on our experience and an updated literature review, the clinical presentation and different diagnostic imaging tools are briefly analysed for each female genital malformation.

1. *Agenesis or hypoplasia of a urogenital ridge*: In these cases, there will be absence of the kidney, ureter, ovary, fallopian tube, hemiuterus and hemivagina (not detectable) on one side (Fig. 2). Clinically, the most common presentation is a unicornuate uterus without a rudimentary horn or contralateral tube and ovary. This condition is sometimes associated with skeletal and/or auditory anomalies [28]. If there is also contralateral Müllerian agenesis, the diagnosis will be Rokitansky syndrome with unilateral renal agenesis [29] or atypical Rokitansky (Fig. 2b). MR is the best diagnostic tool, eventually complemented with hysterosalpingography (HSG) if unicornuate uterus is present. Also, transrectal ultrasound (TRU), i.v. pyelography (IVP) and computed axial tomography (CT) might help. It should be noted that renal agenesis occurs because of lesions of the urogenital ridge and not because of Müllerian agenesis.

**Fig. 2:**
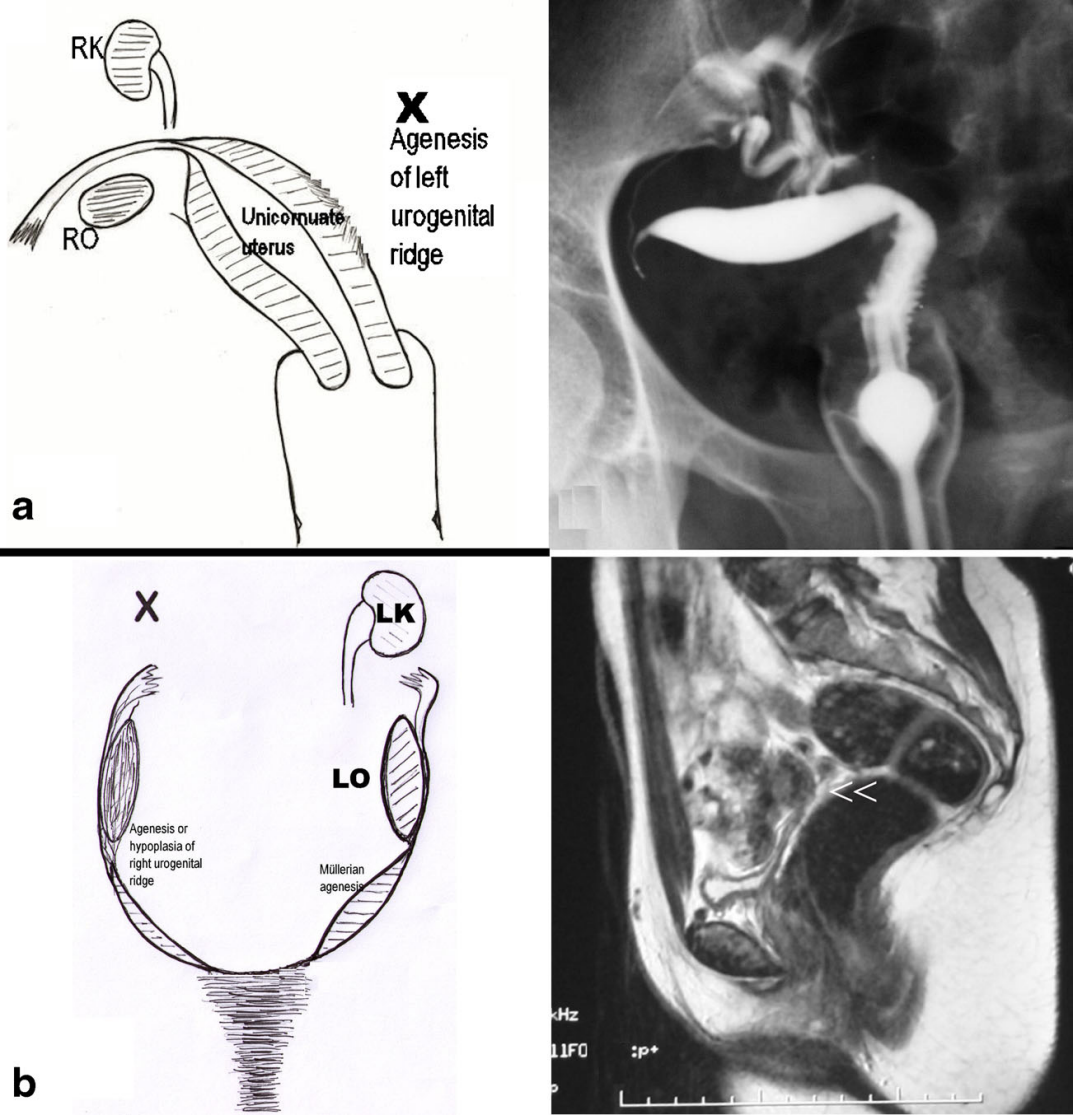
Cases with agenesis or hypoplasia of the urogenital ridge. **a** Schematic representation and HSG showing right unicornuate uterus and agenesis of all derived organs of the left urogenital ridge. **b** Schematic representation and MR in a patient with agenesis of the right urogenital ridge andleft Müllerian duct (Rokitansky syndrome with unilateral renal agenesis). The T2-weighted MR image shows a medial sagittal plane with absence of the uterus and vagina (<<). RO, Right ovary; LO, left ovary; RK, right kidney; LK, left kidney
2. *Distal mesonephric anomalies, including unilateral renal agenesis and ipsilateral blind or atretic hemivagina syndrome*: These are the most complex malformations; they include uterine duplicity (didelphys, bicornuate or less commonly septate uterus), renal agenesis (or dysplasia with or without ectopic ureter) and any of the following subtypes: (a) large haematocolpos in a blind hemivagina, (b) “Gartner’s duct pseudocyst” in the anterolateral wall of the permeable vagina, (c) partial reabsorption of the intervaginal septum or (d) complete unilateral vaginal or cervicovaginal agenesis, with or without communication between both hemiuteri.
  - Cases with unilateral haematocolpos (in girls, hydrocolpos) [30, 31] clinically manifest as progressive intra- and postmenstrual dysmenorrhoea present from menarche. On examination, a lateral and anterior bulge is revealed in the vagina. If haematocolpos is suspected, abdominal, transrectal or transvaginal ultrasound (TVU) can greatly aid the diagnosis, and when IVP and cystoscopy show renal agenesis, the diagnosis is confirmed [15]. Nowadays, an adequately interpreted MR can be conclusive (Fig. 3a). Sometimes, there might be an interuterine communication (at the isthmus level) or intervaginal apex (Fig. 3b). Also, an ectopic ureter opening into the blind vagina can exist [32] and because communication between both sides is common, the symptom is permanent urinary incontinence between normal micturitions. The injection of a contrast agent into the blind hemivagina will allow the identification of the ectopic ureter by retrograde filling [32, 33] (Fig. 3c); 3D-US (Fig. 3d) and MR might be the main diagnostic tools, but the mentioned aspects and the radiographic images after retrograde filling must be considered.

**Fig. 3:**
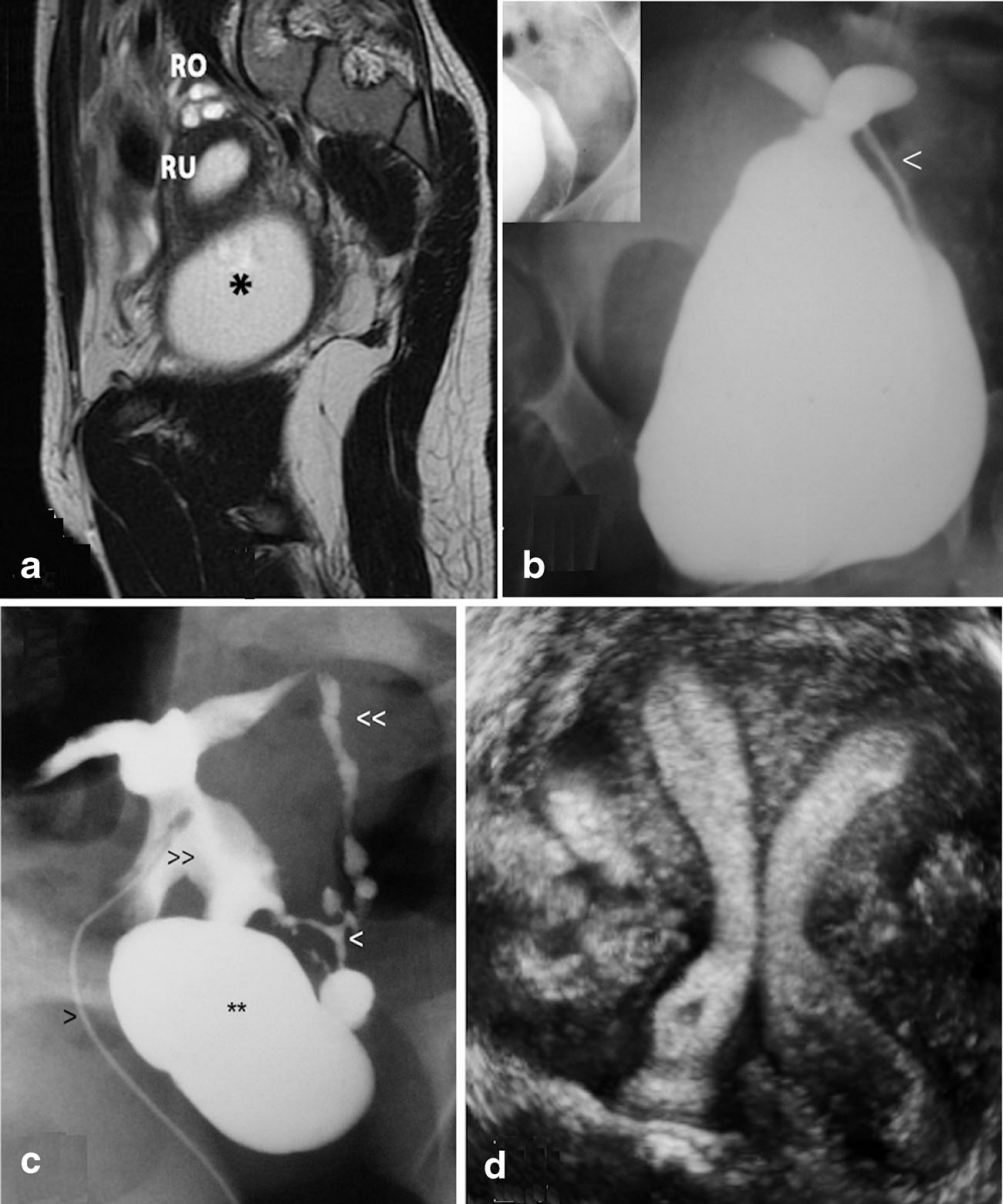
Distal mesonephric anomalies with unilateral blind hemivagina and ipsilateral renal agenesis. **a** MR image corresponding to a 16-year-old patient suffering from strong dysmenorrhoea. After clinical examination and MR, she was diagnosed with endometrioma. However, a dydelphys uterus and right haematocolpos (*) can be observed. T2-weighted MR image, sagittal plane. RO, Right ovary; RU, right hemiuterus (taken from Acién and Acién, Hum Reprod Update 2016;22:48–69, figure 1A1, with permission). **b** An 18-year-old patient presenting with unilateral haematocolpos. Colpo-hysterography after injection of a contrast agent in the right blind hemivagina showing the contrast output through an interuterine communication and left hemivagina (<). **c** Ectopic ureter. HSG image obtained with a small Foley catheter (>) showing the findings in a patient who underwent previous adhesiolysis and Strassman operation abroad. Left blind hemivagina (**), communicating uteri (>>), left ectopic ureter (<<) and possible mesonephric remnants (<) can be observed (modified from Acién et al., Eur J Obstet Gynecol Reprod Biol 2004;117:105–108, with permission). **d** Three-dimensional ultrasound image showing a septate uterus and left blind hemivagina (now perforated) 1 year after drainage of haematocolpos and haematometra (courtesy of Dr. M. Sánchez -Ferrer, Murcia)
  - Patients with “Gartner duct pseudocyst” frequently have no symptomatology other than the fertility problems related to a communicating bicornuate uterus. Examination may reveal a cystic mass with the appearance of a Gartner cyst in the upper anterolateral wall of the vagina. This mass is actually an atretic blind hemivagina [34]. The corresponding hemicervix is usually atretic and the HSG can show a bicornuate-unicollis uterus due to communicating uteri. In other cases it can also be appreciated that the atretic hemicervix is permeable, fistulous and communicates with the atretic blind vagina. These cases correspond with the Herlyn-Werner syndrome [35]. MR and 3D-US could also provide an appropriate diagnosis.
  - Cases with partial reabsorption of the intervaginal septum are similar to the didelphys uterus with a double cervix and vagina, but with unilateral renal agenesis.
  - Cases with complete unilateral vaginal or cervicovaginal agenesis, ipsilateral to the renal agenesis, can have communication between both hemiuteri and will present as a bicornuate-unicollis uterus (communicating uteri). See MR and CT in Fig. 4.

**Fig. 4:**
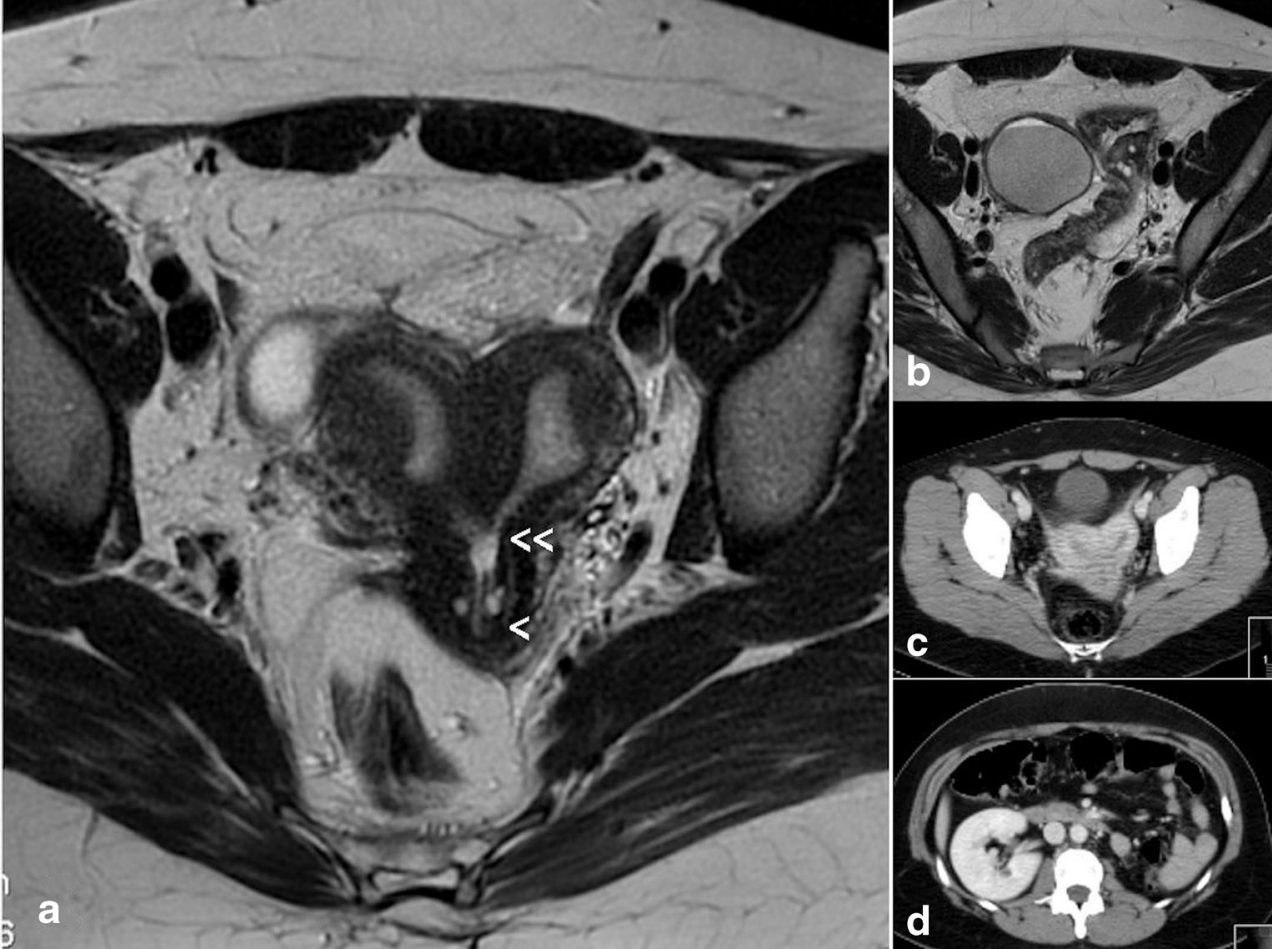
Patient (32 years old) with complete unilateral vaginal or cervicovaginal agenesis or atresia and huge endometrioma. **a** T2-weighted MR image showing a bicornuate (transitional or bicorporeal septate) uterus with communication at the ithsmic level (<<), septate cervix (very thin) with left cervicovaginal atresia (<). At the examination she had only a cervical external os (right side) and also severe endometriosis with great right endometrioma (shown in B). **b** Right endometrioma. **c** CT showing the bicornuate uterus and the cyst (endometrioma). **d** CT showing the left renal agenesis
  - In other cases, there is no communication between the hemiuteri. These cases reflect unilateral haematometra and endometriosis caused by retrograde menstruation on the side of the absent vagina and kidney [36, 37]. Differential diagnosis must be done with Müllerian segmentary atresias [38]. The 2D- and 3D-US, IVP and MR can help in the diagnosis and treatment includes a hemi-hysterectomy [15].

**Fig. 5:**
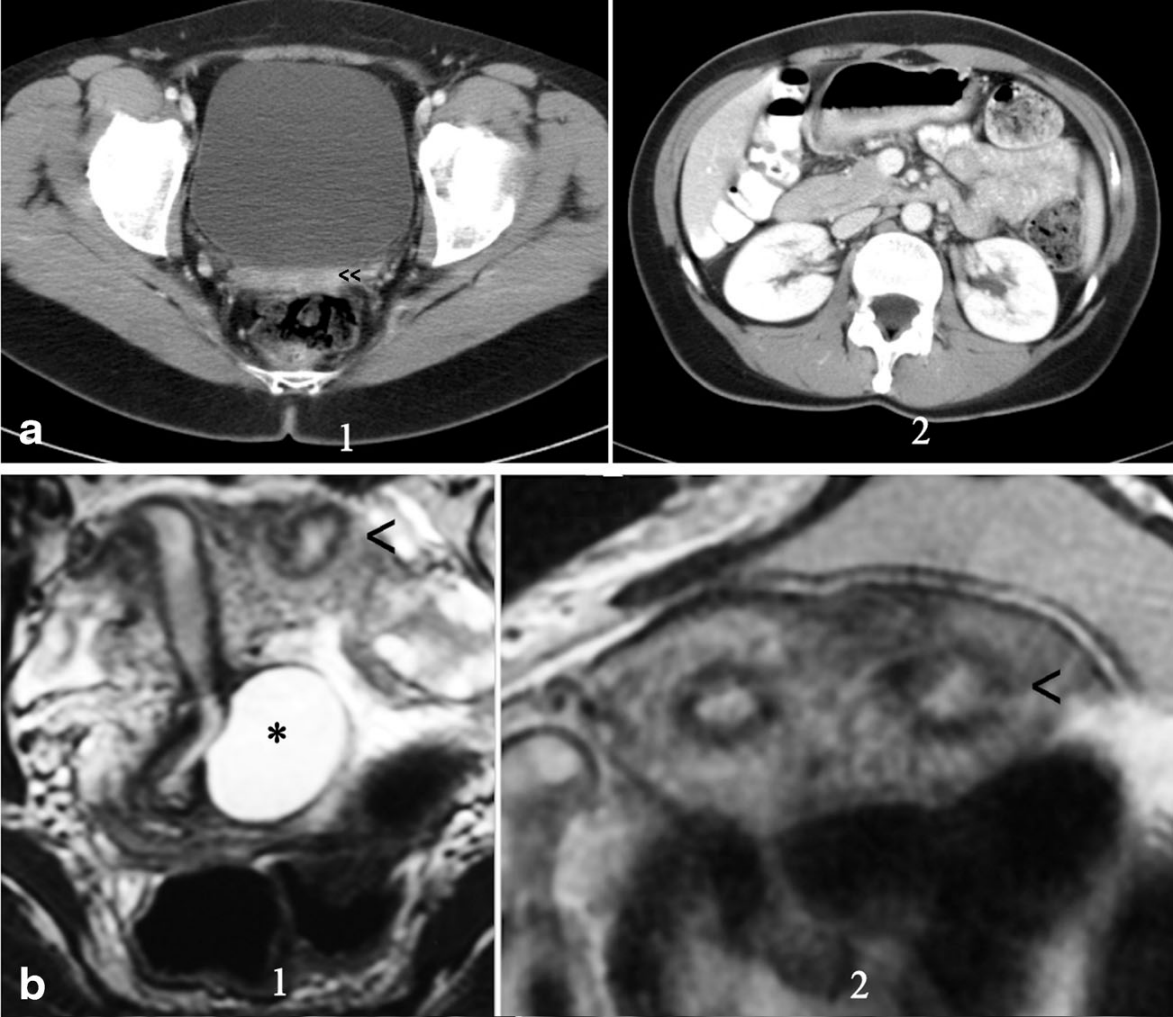
Rokitansky syndrome and unicornuate uterus. **a** (1) CT in a patient with Rokitansky syndrome showing the utero-vaginal rudimentary area under the bladder (<<). (2) Showing both normal kidneys. **b** Patient with a unicornuate uterus and cavitated rudimentary uterine horn. (1) Axial and (2) T2-weighted MR image (coronal cut) showing the left cavitated and rudimentary uterine horn (<). *Left retrocervical subperitoneal serous cyst corresponding to a Müllerian remnant (excised during laparoscopy). (Modified from Acién and Acién, Hum Reprod Update 2016;22:48–69, Fig. 3b, with permission.) The i.v. pyelography showed normal kidneys
  - *Unicornuate uterus* comes in several variations, based on the degree of development and absence of communication to the contralateral side (Fig. 5b). It can be easily diagnosed with HSG, but attention must be given for the possibility of a didelphys uterus with unilateral canalisation and contrast injection. Nowadays, 3D-US is a better tool and MR is of special interest in the detection of a cavitated non-communicated uterine horn, which can also be observed with TVU. It must be remembered that in all isolated Müllerian anomalies both kidneys will be present.
  - *Didelphys uterus* presents two completely detached hemiuteri (like two unicornuate uteri) with two cervices and a double vagina. The new ESHRE/ESGE classification system [22] has assimilated the didelphys to bicornuate uterus, including it as a complete bicorporeal uterus (U3/C2/V2). For the diagnostic imaging, the considerations made on the resorption of the septum and the bidirectional hypothesis of Müller et al. [26] have to be taken into account, and cases with a didelphic uterine corpus and simple (normal or septate) cervix and vagina can be found.
  - *Bicornuate uterus* (Fig. 6a) includes complete (bicornis-bicollis uterus) and partial (bicornis-unicollis uterus) in the AFS/ASRM classification [19] or partial, complete and bicorporeal septate uterus in the new ESHRE/ESGE classification [22]. Some cases can have a cavitated non-communicating horn, and their inclusion as a bicornuate/septate or unicornuate uterus is discussed (see Fig. 5b). Currently, sonohysterography, 3D-US and specially the MR may provide the differential diagnosis with the septate uterus without the need of laparoscopy. In a pelvic MR, a significant fundal cleft (>1 cm) indicates no fusion of the upper-mid uterine horns [19, 46]. However, if this distance measures less than 1 cm, then a septate uterus would be present [46]. In the ESHRE/ESGE classification system [22], class U3 (bicorporeal uterus) is defined by an external indentation of >50 % of the uterine wall thickness, whereas in the complete bicorporeal uterus (U3b), the width of the fundal indentation at the midline is >150 % of the uterine wall thickness.

3. Isolated Müllerian anomalies (without urinary tract anomalies): These include cases of:
  - *Müllerian agenesis*, presenting: (a) vaginal agenesis with a functional uterus, (b) cervical agenesis, (c) uterine fundal or corporal agenesis and (d) tubal agenesis. These are rare anomalies, with 3D-US and MR being highly efficient in the diagnosis of anomalies of the cervix and vagina [39]. However, the combined uterovaginal agenesis is the most common type of agenesis (bilateral Müllerian agenesis) and it corresponds with *MRKH* or *Rokitansky syndrome* [40, 41]. This is an isolated Müllerian anomaly affecting both the Müllerian tubercle and ducts (Fig. 5a). Patients report primary amenorrhoea. TRU, CT or MR [42] demonstrate uterus absence with normal ovaries and two solid rudimentary horns. Some of these rudimentary horns may occasionally present a small functioning endometrial cavity, giving rise to retrograde menstruation and endometriosis [15, 43, 44]. Occasionally, the cavitated rudimentary horn might be well developed, with its reimplantation in a previously performed neovagina being possible [15, 45].

**Fig. 6:**
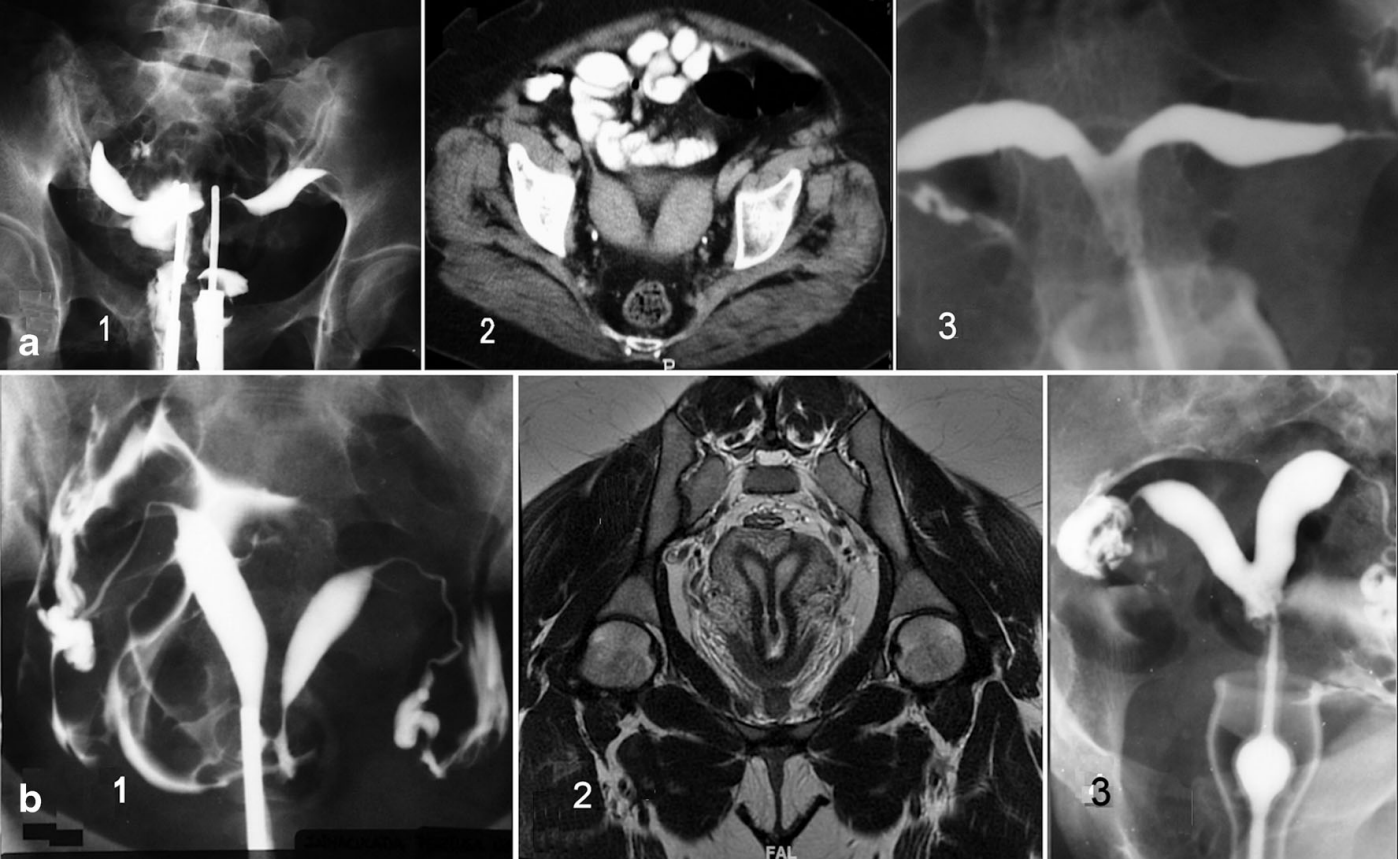
Didelphys, bicornuate and septate uteri. **a** 1. HSG image of a patient with didelphys uterus obtained using a double simultaneous cannula. 2. CT of other patient showing a bicornuate uterus. 3. HSG image of a bicornis-unicollis uterus. **b** *1* HSG showing a complete septate uterus and communicating septate uterus. HSG was obtained using a single cannula through the right side. *2* T2-weighted MR image in a 29-year-old patient showing a septate uterus with septate cervix but single external os and vagina. Coronal plane. *3* HSG of a subseptate uterus
  - *Septate uterus* (complete and partial or subseptate uterus) (Fig. 6b). The diagnosis is equally suggested by TVU and 3D-US or by HSG. Currently, sonohysterography, 3D-US, CT and specially MR can provide the appropriate differential diagnosis [4–7, 47]. Imaging description for septate uterus in the AFS/ASRM classification (class V) is convex, flat or minimally indented (<1 cm) fundal contour with indentation of the myometrium/septum into the uterine cavity (>1 cm) [46]. In the ESHRE classification [22], class U2 (septate uterus) is considered by an internal indentation >50 % of the uterine wall thickness and external contour straight or with indentation <50 % [21, 22].
  - *Arcuate uterus* is a minor form of bicornuate uterus. It has not been included in the new ESHRE/ESGE classification system [22].
  - *Anomalies related to DES syndrome* include hypoplastic, tricavitated and T-shaped hypoplastic uteri with an extremely small uterine cavity, cornual constrictions and bulbous dilatation of the lower segment. In the new ESHRE/ESGE classification [22], these anomalies are included as dysmorphic uterus (class U1).
  - Isolated Müllerian anomalies affecting the Müllerian tubercle include: (1) complete vaginal (or cervicovaginal) agenesis or atresia and (b) segmentary atresias, as in cases of transverse vaginal septum.
    Complete vaginal or *cervicovaginal agenesis or atresia with a functional uterus* is usually a complex malformation in which the external genitals and tubes appear normal. The uterus may be normal or may present with fusion or resorption defects and the cervix may be present, absent or hypoplastic. The clinical presentation involves primary amenorrhoea and cyclic pain in postpubertal women. TRU and particularly MR (Fig. 7) allow a clear diagnosis that includes a largely normal corpus uteri with endometrium and cervicovaginal atresia. The ovaries are normal, although they might present endometriosis because of retrograde menstruation. Laparotomy with atretic cervix resection and reimplantation of the uterine corpus in the neovagina is recommended, having achieved normal menstruations and spontaneous term pregnancy [15, 48].

**Fig. 7:**
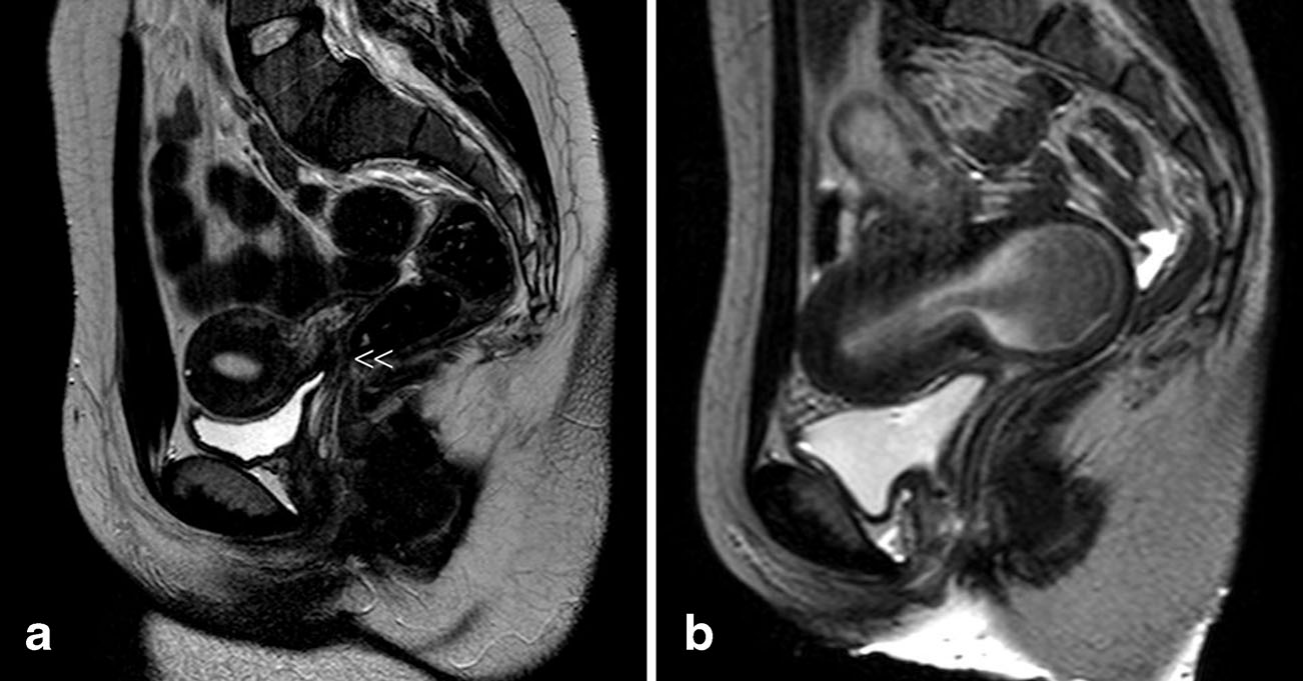
Cases with vaginal or cervico-vaginal atresia or agenesis and normal uterine corpus. **a** T2-weighted MR image in a 20-year-old patient with complete cervico-vaginal atresia. Medial sagittal section showing the uterus and cervico-vaginal atresia (<<). **b** T2-weighted sagittal plane in the other case with vaginal atresia and haematocervicometra (courtesy of Dr. MJ. Lázaro, Oviedo)
    *Vaginal segmentary atresia and transverse vaginal septum* correspond to a transverse constriction or septum that is perforated or imperforated. There may be no symptoms until puberty when the haematocolpos forms and causes episodes of pelvic pain and primary amenorrhoea similar to those observed with vaginal atresia [49, 50]. The examination, abdominal or TRU and specially MR allow the diagnosis and help on the surgical evacuation of the haematocolpos. Uterus, fallopian tubes and ovaries are usually normal.

**Fig. 8:**
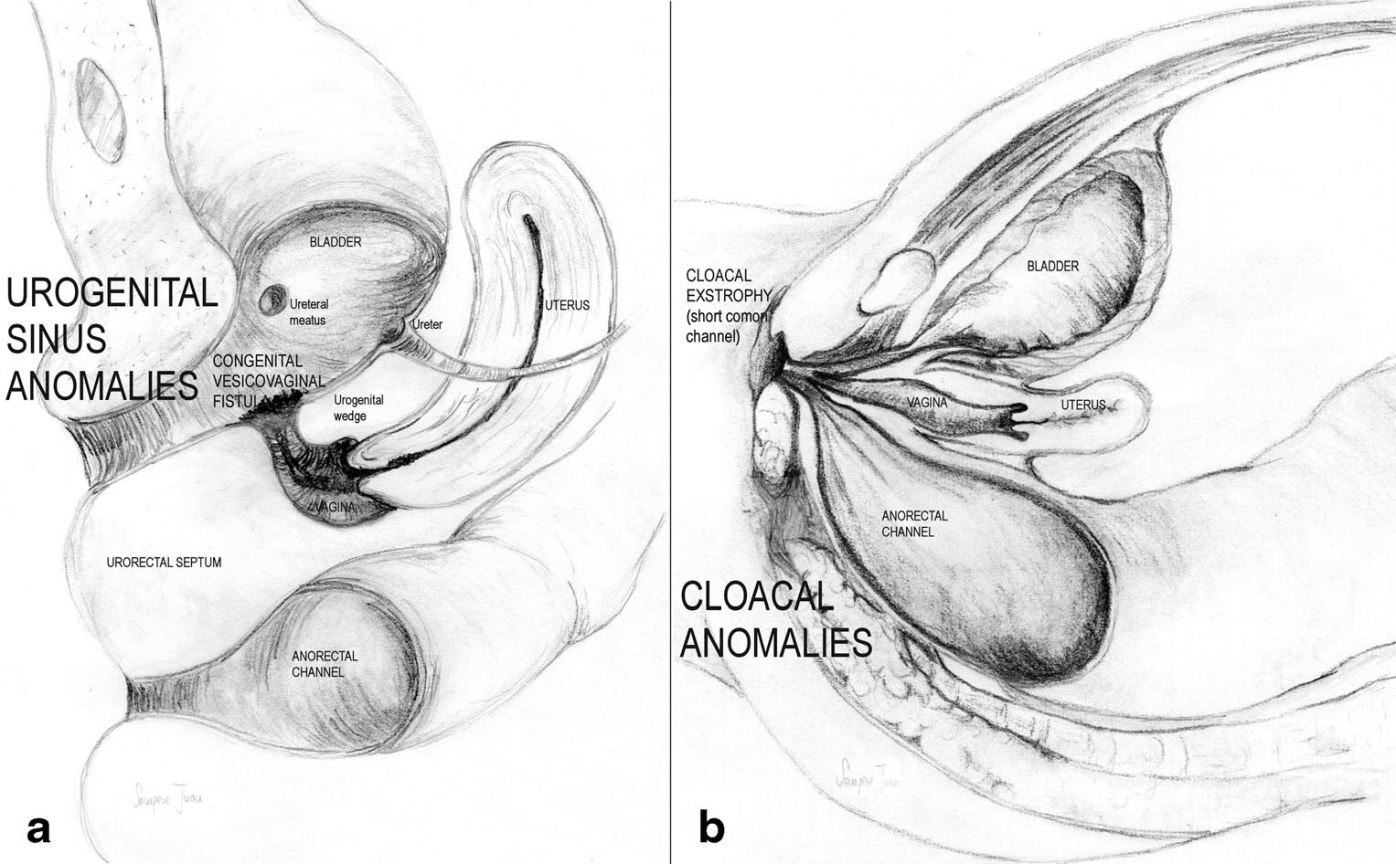
Schematic representation of urogenital sinus and cloacal anomalies. **a** Congenital vesico-vaginal (pseudo)fistula. **b** Cloacal malformations: cloaca with a short common cannel
  A *congenital vesicovaginal fistula* is a rare, complex female genital malformation that is difficult to diagnose, classify and treat. Its embryological origin lies in the abnormal persistence of the urogenital sinus due to the lack of formation and caudal growth of the urogenital wedge [8, 15]. This diagnosis should be suspected in any girl with urinary incontinence, urinary tract infections from birth, vaginal swelling or hydrocolpometra and in adults with cyclical menouria and vaginal atresia [65]. Foetal urinary ascites and hydrometrocolpos might be a consequence of persistent urogenital sinus and result of a vesicovaginal fistula [66, 67]. The diagnosis should be based on a high index of suspicion in second trimester US and an MR in the third trimester of pregnancy. However, in adolescent or adult women, the diagnosis should also be based on suspicion, but especially on physical examination, cystoscopy during menouria and imaging (US and MR as shown in Fig. 9) [61].

4. *Gubernaculum dysfunctions*: These cases are typified by accessory and cavitated uterine masses (ACUMs) with an otherwise normal uterus [51–53]. HSG will show a normal endometrial cavity and 3D-US and especially MR allow the right diagnosis.
5. *Anomalies of the cloaca and urogenital sinus (including congenital vagino-vesical fistulas)*: This category includes cases as simple as the imperforate hymen due to a persistent urogenital membrane together with blind hemibladder [54], bladder duplication [55, 56], bilateral single system ectopic ureters opening into the vestibule or a vaginalised urogenital sinus with bladder agenesis or hypoplastic bladder [57–59] or congenital vesico-vaginal or vesico-uterine fistulas (pseudofistula with menuria [60, 61]) and cloacal exstrophy [62, 63] (Fig. 8). Diagnosis can be made with the physical examination together with TRU, IVP (eventually, retrograde pyelogram), cystouretroscopy, cystography, CT, and specially MR, or abdominal US for prenatal diagnosis [56, 64].

**Fig. 9:**
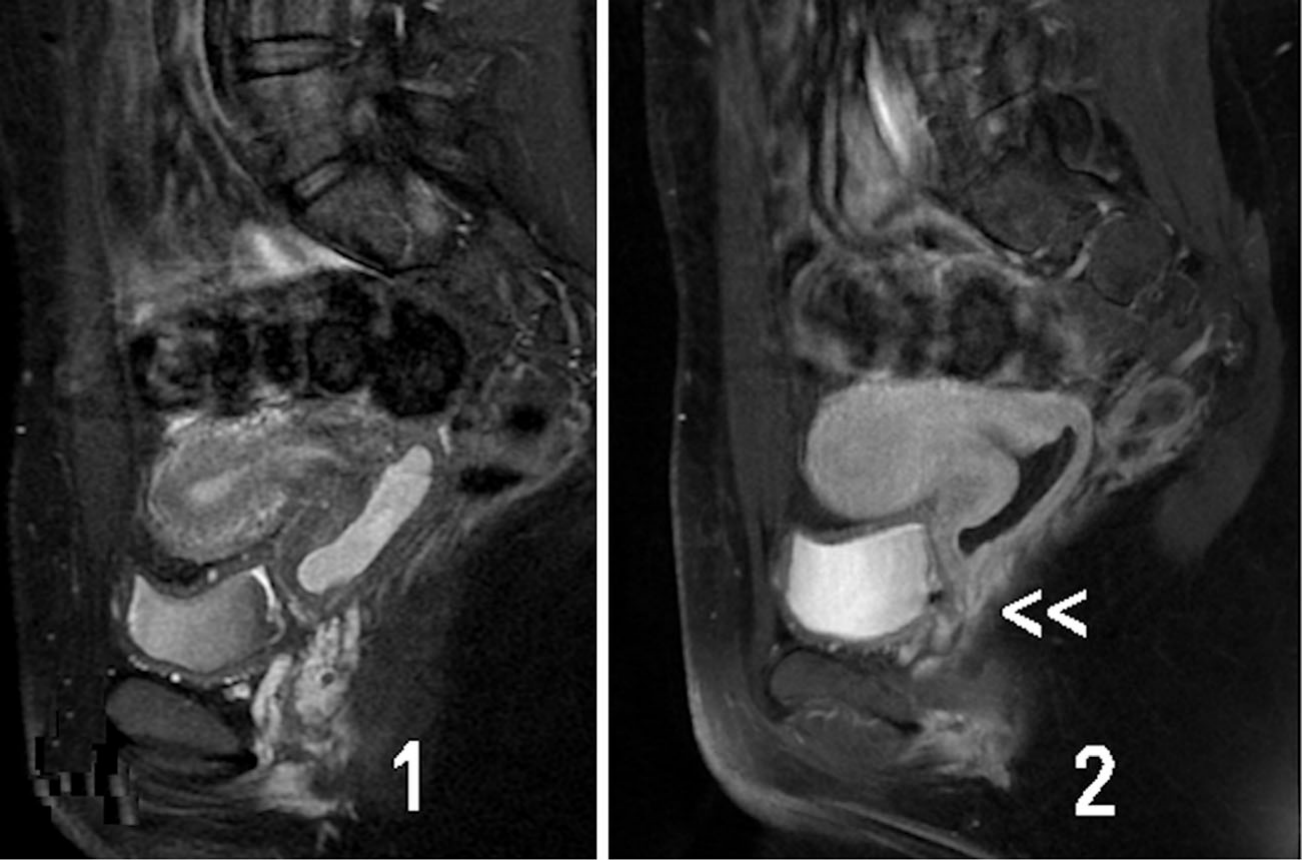
Urogenital sinus anomalies. MR images [(1) T1- and (2) T2-weighted fat-supressed MR in the sagittal plane] showing a retrovesical blind vagina with apparent inferior half atresia and undetected fistula tract to the bladder (<<) in a 28-year-old patient. Cystoscopy confirmed the presence of an orifice situated in the trigone, just above the bladder neck, equidistant and below both ureteral meati, through which menstrual blood clearly exited from the vagina. The patient suffered from cyclic menuria and the opening of the fistulous tract into the bladder trigone was in fact the hymen (courtesy of Dr. JC. Martínez-Escoriza, Alicante)
  A *rectovestibular fistula* often coexists with vaginal or vestibular atresia. Female cloacal exstrophy occurs when the urorectal septum fails to separate from the cloacal membrane, resulting in the urethra, vagina, rectum and anus opening into a single common channel (Fig. 8b). MR could accurately demonstrate the level of bowel termination in patients with persistent cloaca in addition to its high sensitivity for detection of Müllerian anomalies, which are present in 73 % of patients [68].
6. *Malformative combinations*: Some patients may present several associated anomalies of mesonephric, Müllerian and/or cloacal origin [9, 54, 69] that result in very complex malformations [70, 71] with a rich chart of symptoms that may be difficult to appropriately catalogue and treat, especially if the embryology and physiopathology of the female genital tract is not taken into consideration.

## Conclusions

1. The combination of uterine duplicity and obstructed or blind hemivagina appears to be virtually always associated with ipsilateral renal agenesis or dysgenesis. Imaging tests (IVP, MR) are necessary not only to confirm the absence of a normal kidney on the affected side, but also to detect abnormalities of the contralateral kidney and/or ureter [72].
2. Although cases with normal kidneys have also been described [73, 74], the analysis of what is referred to in these papers shows that there was always some kind of reno-ureteral anomaly or malrotation [75]. Furthermore, if there is unilateral renal agenesis, there must also be genital malformation with didelphys, bicornuate and more rarely septate uterus (sometimes reported as single uterus [76]), but not necessarily a blind or atretic hemivagina [54, 77]. There could also be cases with partial reabsorption of the vaginal septum, but no case has been reported in the literature with unilateral renal agenesis, normal uterus and vagina.
3. Another controversial point is the differentiation between a bicornuate and a septate uterus. However, the distinction is very important for the treatment of symptomatic patients (abdominal Strassmann metroplasty on the bicornuate uterus versus hysteroscopic resection in cases of septate). TVU, 3D-US and sonohysterography, especially MR, have ushered in a new era of non-invasive diagnosis of uterine anomalies [4–7, 20, 47, 78]. Nevertheless, there are transitional cases between both uterine segments in which fusion defects are associated with resorption defects and these situations must also be recognised before a therapeutic decision can be made.

## Abbreviations

US: Ultrasound (two- and three-dimensional)
CT: Computed axial tomography
MR: Magnetic resonance image
HSG: Hysterosalpingography
IVP: i.v. pyelography
TVU: Trasvaginal ultrasound
TRU: Transrectal ultrasound
ASRM: American Society for Reproductive Medicine
MRKH: Mayer-Rokitansky-Kuster-Hauser
ESHRE/ESGE: European Society for Human Reproduction and Embryology/European Society for Gynaecological Endoscopy
